# Effect of number of medications and use of potentially inappropriate medications on frailty among early-stage older outpatients

**DOI:** 10.1186/s40780-021-00195-x

**Published:** 2021-05-03

**Authors:** Yuya Uragami, Kazuhiro Takikawa, Hajime Kareki, Koji Kimura, Kazuyuki Yamamoto, Naomi Iihara

**Affiliations:** 1 Star Pharmacy Co., Ltd, 4113–1 Onohara Onohara-cho, Kagawa 769–1611 Kanonji City, Japan; 2 Kagawa School of Pharmaceutical Sciences, Tokushima Bunri University, 1314–1 Shido, Sanuki City, Kagawa 769–2193 Japan

**Keywords:** Frailty, Polypharmacy, Elderly

## Abstract

**Background:**

Frailty is an urgent concern among an aging population worldwide. However, the relationship between frailty and number and types of medications has not been studied in detail among early-stage older patients, and it is unclear what prescriptions may have a role in preventing frailty. This study aimed to clarify the effects of number of medications and use of potentially inappropriate medications (PIMs) on frailty among early-stage older outpatients in Japan.

**Methods:**

A cross-sectional study was undertaken. Frailty scores and medications of outpatients aged 65–74 years who regularly visited community pharmacies were investigated. Frailty scores were classified as 0 (non-frailty), 1–2 (pre-frailty), and ≥ 3 (frailty). The association between frailty and number of medications was analyzed by age and compared between PIM use and non-use groups. The proportion of patients who used PIMs was also analyzed by frailty score.

**Results:**

Of 923 older outpatients, 49 (5.3%) and 318 (34.5%) patients had frailty and pre-frailty scores, respectively. The numbers of medications among patients with pre-frailty and frailty were significantly higher than among those with non-frailty (*p* <  0.001 for both). A similar increase was shown for PIM use groups aged 69–71 and 72–74 years, but not for the PIM use group aged 65–68 years and all groups without PIM use. An increasing linear trend was observed for the relationship between the proportion of patients who used any PIM, as well as some subcategories of PIMs (such as NSAIDs, benzodiazepines, loop diuretics and antithrombotic drugs) and frailty score.

**Conclusions:**

Unnecessary medication use among early-stage older outpatients, especially patients aged ≥69 years who use PIMs and many medications, seems to be associated with frailty, but further research is needed to confirm these findings.

**Supplementary Information:**

The online version contains supplementary material available at 10.1186/s40780-021-00195-x.

## Background

Frailty is defined as decreased physiologic reserves and increased vulnerability to adverse health outcomes among older adults [1]. Frailty is known to be associated with an increased risk of functional limitations, falls, hospitalizations, and death [2], resulting in increased use of healthcare resources [3]. Therefore, countermeasures to prevent and treat frailty are of urgent concern in an aging world.

Numerous factors trigger frailty, including nutrition [4], physical activity [5], and various diseases [6]. Some cross-sectional and longitudinal studies [7–9] reported that potentially inappropriate medications (PIMs), defined by criteria such as the Beers criteria [10] and the Screening Tool of Older Person’s Prescriptions (STOPP) criteria [11], including anticholinergic [7, 8] and sedative drugs [7, 9], pose a risk of frailty in older people. In addition, the number of medications used by older people [12] is known to be associated with frailty.

The Japan Geriatrics Society published *Guidelines for Safe Pharmacotherapy for the Elderly 2005* as an alternative to the Beers and STOPP criteria, and the guidelines reflected the medical and medication situation in Japan; the document was revised as the *Screening Tool for Older Person’s Appropriate Prescriptions* for Japanese (STOPP-J) in 2015 [13]. Only one study, which was conducted among older patients with mild dementia, analyzed the association between PIMs listed in STOPP-J and frailty in Japan [14]. The authors did not find any association between PIMs and frailty, but they did show an association between frailty and reduced quality of life and verbal fluency. Thus, the relationship between frailty and use of PIMs has not been well studied in Japan, although an association between frailty and number of medications has already been found in several studies [15–17].

The number patients with frailty increases greatly after age 75 [18]. To prevent the onset of frailty and control its progression, a study targeting early-stage older people is needed. The present study aimed to clarify the effects of the number of medications and use of PIMs on frailty among early-stage older outpatients in Japan by using STOPP-J.

## Methods

### Study population

This cross-sectional study was undertaken in outpatients aged 65–74 years who (1) visited any of 11 community pharmacies of the Star Pharmacy Group in Kagawa Prefecture in Japan from February to April 2020, (2) visited the same community pharmacies regularly for at least three months before entry into the present study, and (3) provided written informed consent for the study. Those who were certified to need any nursing care support were excluded, because the simple sheet used to measure frailty in the present study, as mentioned below, was developed for patients not receiving nursing care support [19].

The community pharmacies are located near general hospitals and clinics and handle a wide range of medication classes, including those for cardiology, gastroenterology, respiratory, pediatrics, and orthopedics. The study protocol was approved by the ethics committee of Tokushima Bunri University in December 2019 (No. R1–37).

### Survey

Pharmacists in the community pharmacies measured the frailty of participants using a simple sheet developed by Kumagai [19]. The sheet, which has been validated [19], is a questionnaire that has been adapted from the Japanese version of the Fried frailty phenotype [18] to enable healthcare providers to measure frailty easily. The questionnaire consists of five items (fatigue, resistance, ambulation, inactivity, and loss of weight) with response choices of “yes” or “no”; the total score ranges from 0 (no frailty) to 5 (extreme frailty). A total score (frailty score) of ≥3 is considered to indicate frailty. The frailty scores in the present study were grouped into three levels (0, 1–2, and ≥ 3), since the simple sheet by Kumagai [19] was developed based on the Cardiovascular Health Study criteria published by Fried [6], and those criteria employ levels of 0 (non-frailty), 1–2 (pre-frailty), and ≥ 3 (frailty).

Pharmacists also investigated the medications that the participants had been using regularly for more than four weeks. Concomitant medications were investigated by using a prescription notebook that patients carried to the pharmacy and pharmacists completed. These medications included oral medications and insulin products and did not include medications that were used on an as-needed basis or over-the-counter drugs.

The investigated medications were classified as PIMs and not PIMs. PIMs were defined using STOPP-J, and were classified into 19 categories and 28 subcategories (Supplementary Table 1); the categories consisted of antipsychotics, hypnotics, antidepressants, sulpiride, antiparkinson drugs, steroids, antithrombotic drugs (antiplatelet drugs and anticoagulants), digitalis, diuretics, β-blockers, α-blockers, first-generation H_1_ receptor antagonists, H_2_ receptor antagonists, antiemetics, laxatives, antidiabetic drugs, insulin, overactive bladder medications, and nonsteroidal anti-inflammatory drugs (NSAIDs). Antipsychotics, selective serotonin reuptake inhibitor (SSRI) antidepressants, steroids, digitalis, β-blockers, and laxatives are considered PIMs in STOPP-J only in certain cases or in certain subsets of patients (e.g., use of laxatives among patients with impaired renal function), but the present study considered these PIMs in any patients.

### Statistical analyses

The following items were evaluated: (1) gender and age by frailty score level, (2) number of medications by frailty score level, (3) proportion of patients who used PIMs according to frailty score level and (4) number of medications according to frailty score level in the PIM use group (PIMs group) and PIM non-use group (non-PIMs group). The PIMs group included those who used at least one PIM.

Participants were divided into three age subgroups by tertile: 65–68, 69–71 and 72–74 years of age, to try to obtain a similar number of patients per subgroup. The relationship between frailty score and the number of medications was assessed for all participants and for these age subgroups. The number of medications was defined as the number of all investigated medications. To investigate the proportion of patients who used PIMs according to frailty score levels, we calculated frailty levels with any PIM and in every subcategory of PIM. Calculating the proportion for each subcategory, we first estimated the correlation coefficients between frailty score level and PIM use for each subcategory and next calculated the proportion of patients who used PIMs, only if the subcategories had a significant correlation coefficient and included 10 or more patients.

The chi-square test for nominal variables and the Cochran-Armitage trend test for ordinal variables were used. The Kruskal-Wallis test and Holm’s multiple test were used to compare continuous variables among the three groups. The significance level was established at 0.05. EZR version 1.37 (Saitama Medical Center, Saitama, Japan) [20] was used for statistical analyses.

## Results

Of 926 patients who were informed about the present study, 923 (99.7%) provided consent and were enrolled in the study. The population included 432 males (46.8%) and 491 females (53.2%), and the median age of the entire group was 70 years. In all, there were 49 patients (5.3%) with frailty (frailty score ≥ 3), 318 patients (34.5%) with pre-frailty (frailty score 1–2), and 556 patients (60.2%) with non-frailty (frailty score 0). Concomitant medications were identified in 255 patients (27.6%) based on the information in their prescription notebooks.

Gender and age by frailty score level are shown in Table 1. There was no difference in gender distribution among patient groups with frailty score levels of 0, 1–2, or ≥ 3 (*p* = 0.132). However, there was a difference in the proportion of patients included in each age subgroup (*p* = 0.025); the proportion of patients in the 65–68-years age subgroup was likely to be lower among the pre-frailty or frailty group than the non-frailty group.

**Table 1:** Gender and Age by Frailty Score Level

Variables	Frailty score 0 *n* = 556 *n* (%)	Frailty score 1–2 *n* = 318 *n* (%)	Frailty score ≥ 3 *n* = 49 *n* (%)	*p-*value^a^
Gender Male	275 (49.5)	135 (42.5)	22 (44.9)	0.132
Female	281 (50.5)	183 (57.5)	27 (55.1)	
Age, median (interquartile range)	70 (68, 72)	71 (68.25, 72)	70 (68, 72)	
65–68 years	197 (35.4)	80 (25.2)	14 (28.6)	0.025
69–71 years	193 (34.7)	119 (37.4)	18 (36.7)	
72–74 years	166 (29.9)	119 (37.4)	17 (34.7)	

^a^ Chi-square test

**Fig. 1:**
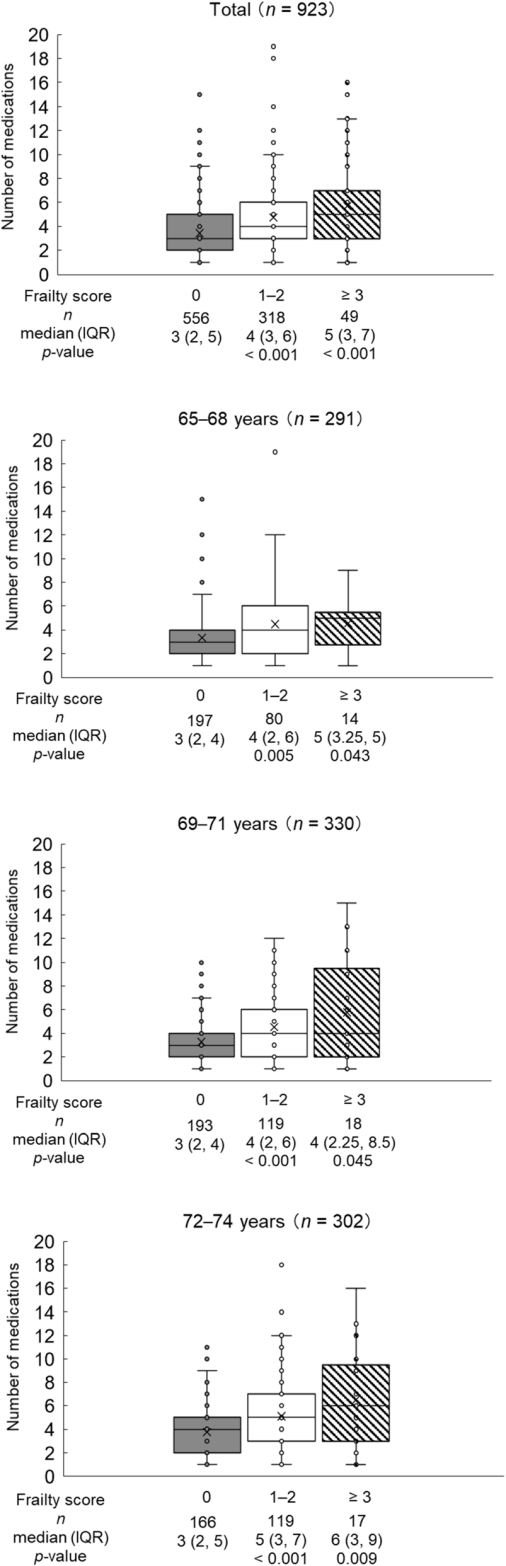
Number of Medications by Frailty Score Level. IQR interquartile range

The number of medications by frailty score level is shown in Fig. 1. For all of the early-stage older patients, the number of medications was higher among those with a frailty score of 1–2 (*p* <  0.001) and ≥ 3 (*p* <  0.001) than among those with a frailty score of 0; the median (interquartile range) numbers of medications for those with a frailty score of 0, 1–2, and ≥ 3 were 3 (2, 5), 4 (3, 6), and 5 (3, 7). In addition, a higher number of medications with the increase in frailty scores was shown for age subgroups (Fig. 1).

The proportions of patients who used PIMs according to frailty score level are shown in Table 2. The proportion of patients who used any PIM increased with increasing frailty score (*p* <  0.001); the proportions among frailty score levels 0, 1–2, and ≥ 3 were 37.4% (208/556 patients), 56.0% (178/318 patients), and 69.4% (34/49 patients), respectively.

**Table 2:** Proportion of Patients who Used PIMs by Frailty Score Level

PIM use	Total *n* = 923 *n* (%)	Frailty score 0 *n* = 556 *n* (%)	Frailty score 1–2 *n* = 318 *n* (%)	Frailty score ≥ 3 *n* = 49 *n* (%)	*p-*value^a^
Any PIM	420 (45.5)	208 (37.4)	178 (56.0)	34 (69.4)	< 0.001
PIMs					
NSAIDs	71 (7.7)	24 (4.3)	38 (11.9)	9 (18.4)	< 0.001
Benzodiazepines^b^	90 (9.8)	40 (7.2)	40 (12.6)	10 (20.4)	< 0.001
Loop diuretics	16 (1.7)	3 (0.5)	12 (3.8)	1 (2.0)	0.004
Antithrombotic drugs					
Antiplatelet drugs including aspirin	84 (9.1)	36 (6.5)	44 (13.8)	4 (8.2)	0.007
Combined therapy with multiple antithrombotic drugs	21 (2.3)	7 (1.3)	13 (4.1)	1 (2.0)	0.04
Muscarinic receptor antagonists	26 (2.5)	10 (1.8)	12 (3.8)	4 (8.2)	0.006
H_2_ receptor antagonists	30 (3.3)	12 (2.2)	15 (4.7)	3 (6.1)	0.02
Sulfonylureas	39 (4.2)	17 (3.1)	18 (5.7)	4 (8.2)	0.021
Thiazolidine derivatives	11 (1.2)	3 (0.5)	7 (2.2)	1 (2.0)	0.039
SSRIs	12 (1.3)	4 (0.7)	6 (1.9)	2 (4.1)	0.025

^a^ Cochran-Armitage trend test

^b^ Including use as antianxiety drugs and hypnotics

NSAIDs non-steroidal anti-inflammatory drugs; PIMs potentially inappropriate medications; SSRIs selective serotonin reuptake inhibitors

The correlations between PIM use of each subcategory and frailty score were weak, although some subcategories showed significant correlations (Supplementary Table 1). PIMs that had the highest correlation coefficients were NSAIDs (0.162, *p* <  0.001), benzodiazepines (0.114, *p* <  0.001), loop diuretics (0.106, *p* = 0.001), and antiplatelet drugs including aspirin (0.103, *p* = 0.002), followed by muscarinic receptor antagonists, multiple antithrombotic drugs, H_2_ receptor antagonists, sulfonylureas, thiazolidine derivatives and SSRIs. Linear trends for the proportion of patients who used PIMs by increasing frailty score are shown for the PIM subcategories described above (Table 2).

**Fig. 2:**
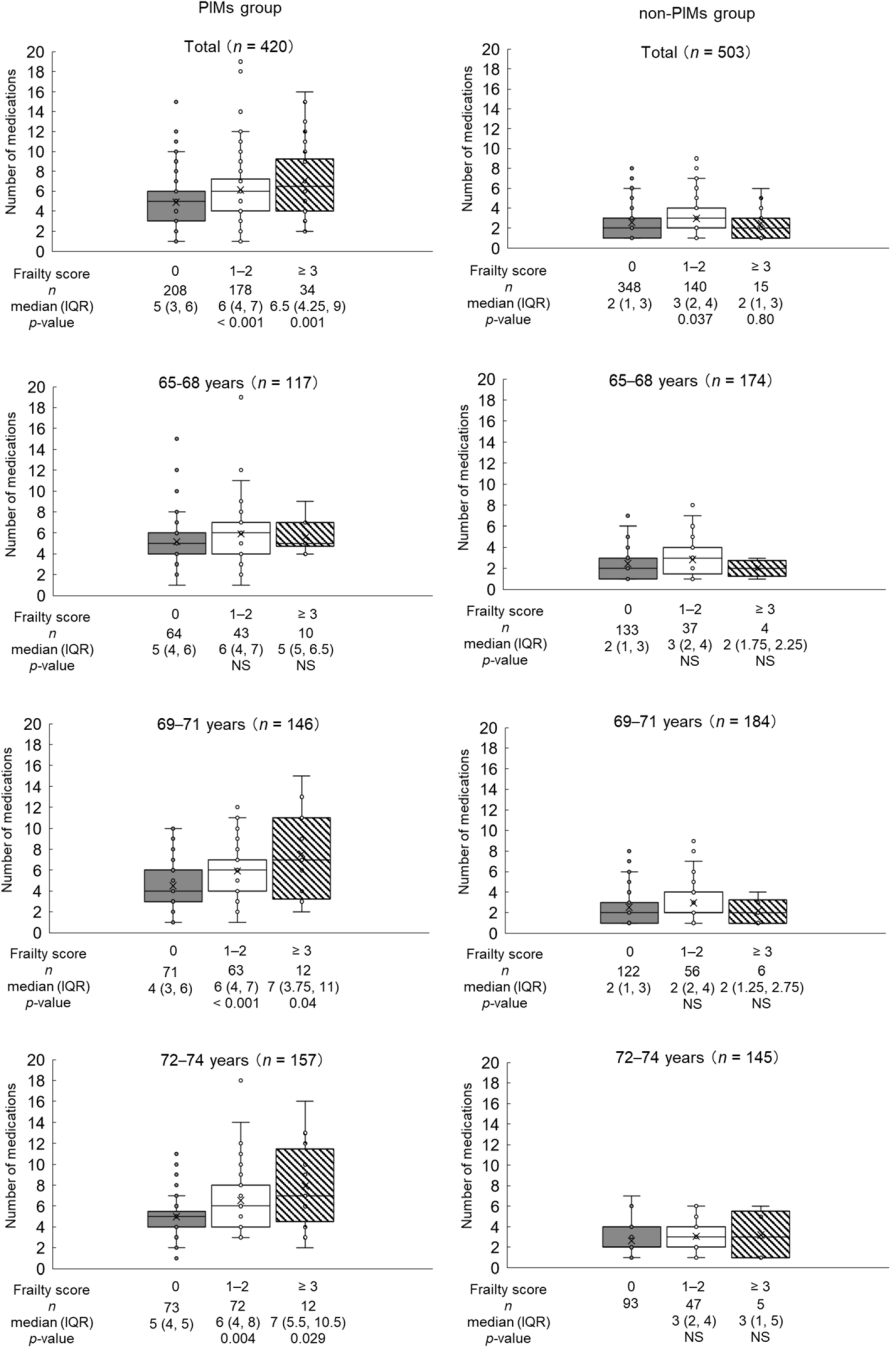
Number of Medications by Frailty Score Level in the PIMs and Non-PIMs Groups. IQR interquartile range; PIM potentially inappropriate medication; PIMs group PIM use group; non-PIMs group PIM non-use group

The PIMs and non-PIMs groups included 420 patients (45.5%) and 503 patients (54.5%), respectively. Figure 2 shows the number of medications according to frailty score level in the PIMs and non-PIMs groups. Considering all patients in the PIMs group, the number of medications increased with increasing frailty score; the median (interquartile range) numbers for frailty score levels of 0, 1–2, and ≥ 3 were 5 (3, 6), 6 (4, 7), and 6.5 (4.25, 9), respectively, and significant differences in the number of medications were observed for comparisons between frailty score levels of 0 and 1–2 (*p* < 0.001) and between 0 and ≥ 3 (*p* = 0.001). On the other hand, for all patients in the non-PIMs group, there were significant differences observed for comparisons between frailty score levels 0 and 1–2 (*p* = 0.037) but not between 0 and ≥ 3 (*p* = 0.80).

An increase in number of medications according to increased frailty score level was seen for the 69–71-years and the 72–74-years groups in the PIMs group, but no increase was observed for the 65–68-years age subgroup in the PIMs group and all age subgroups in the non-PIMs group (Fig. 2).

## Discussion

The present study evaluating early-stage older outpatients found an association between an increased number of medications and increased frailty score level; this association was noticeable among patients aged 69–74 years in the PIMs group but not in the non-PIMs group. Therefore, patients aged ≥69 years who use PIMs and many medications may need to be closely monitored for unnecessary and inappropriately prolonged medication use.

The findings of the present study require careful interpretation. This study was a cross-sectional study. In addition, some existing reports show an association between PIM use and number of medications [9, 21] and an association between the number of medications and frailty [12]. Besides, numerous factors trigger frailty, such as nutrition [4], physical activity [5], and various diseases [6]. Thus, we cannot assume a direct causal relationship between the use of PIMs and many medications and frailty for early-stage older outpatients. We have not found reports that state whether correcting inappropriate prescriptions, including reducing the number of medications, leads to prevention of frailty. However, approximately 70% of patients who take 5 or more medications and approximately 80% of patients who take 10 or more medications are older than 65 years [22], and the number patients with frailty increases greatly after age 75 in Japan [18]. Therefore, further research is needed to confirm these findings and to analyze whether reducing the number of medications and reviewing the use of PIMs from the early stage of older age can help prevent the onset of frailty and control its progression.

On the other hand, polypharmacy appears to affect frailty, and frailty may also affect polypharmacy, as health problems resulting from frailty may lead to a need for additional medications, and diseases associated with frailty may lead to polypharmacy as well. Thus, since prevention of frailty may help to avoid polypharmacy, healthcare providers should strive to prevent frailty to minimize the likelihood and adverse effects of polypharmacy.

The present study found some associations between PIM subcategories and frailty. Subcategories such as NSAIDs [23], benzodiazepines [7, 9], muscarinic receptor antagonists [7, 8], and SSRIs [24] have been associated with frailty in previous studies. For medications in other subcategories, we could not find reports of associations with frailty, so it is unclear whether the medications are the direct cause of the frailty. However, adverse drug events related to the medications cause frailty: falls with loop diuretics [25] or sulfonylureas [26], bone loss with loop diuretics [27], cognitive decline with H_2_ receptor antagonists [28], and osteoporosis with thiazolidine derivatives [29]. Additionally, pathophysiology and conditions that accompanied the use of the medications may have an effect on frailty; for example, diabetes mellitus, pulmonary diseases, and cardiovascular disease are known to be associated with frailty [6]. No report of a direct or indirect effect of antiplatelet drugs on frailty exists, as far as we could determine from searches of the literature.

The present study found the association between number of medications and frailty in all age subgroups of early-stage older patients. Previous studies [8, 30–37] found an association between increased number of medications and frailty for people 65 years of age and older, but all of the previous studies included late-stage older patients: they did not target only early-stage older patients and did not analyze a subgroup of early-stage older age. The present study has an advantage since the association was proven for the early-stage older population.

Some limitations must be considered for the present study. First, the survey was conducted in community pharmacies in Kagawa Prefecture in Japan. However, the median number of medications among patients with frailty was 5 in the entire population and 6.5 in the PIMs group, similar to the results of a previous study that reported the use of 6 or more medications was a high risk for frailty [38]. Second, we are aware that the cutoff points of the age subgroups and the small sample sizes of the groups may have influenced the results. We tried to divide the number of patients into age subgroups of similar size using tertiles and to find an appropriate age cutoff that made the number of patients more even by moving the cutoff value of one tertile to the next age subgroup, but we could not achieve an equal number of patients among the subgroups. Thus, the age cutoff point itself does not have any specific implication, such as on biology and clinical pharmacology. Third, medication dose and laboratory data of renal and hepatic function affect outcomes of medication therapy and might possibly affect frailty, but that information was not analyzed in the present study. Additionally, concomitant medications were investigated in the present study based on information recorded by a pharmacist in a prescription notebook carried by the patient, but the medications may not have been accurately recorded, and adherence to those medications was unclear.

Fourth, the present study counted PIMs without restriction of patients, although STOPP-J [13] restricts patients for some subcategories of PIMs. Thus, the present study may have overestimated exposure to PIMs. Fifth, a seasonal bias for frailty evaluation may exist, since the patient entry period in the present study was limited to three months, and older people are considered to be likely to exercise less and to stay indoors in the summer and winter.

## Conclusions

The present study targeting early-stage older outpatients found that the association between an increased number of medications and frailty was observed among outpatients aged 69 years and older who used PIMs, but not among those who did not use PIMs. Further research is needed to confirm these findings and analyze whether correcting inappropriate prescriptions, including reducing the number of medications, from the early stage of older age can help prevent the onset of frailty and control its progression.

## Supplementary Information

**Additional file 1: Supplementary Table 1**. Correlation Coefficients between PIM Use and Frailty Score Level. We estimated the correlation between frailty score level and PIM use for each subcategory.

## Data Availability

The datasets used and/or analyzed during the current study are available from the corresponding author on reasonable request.

## Abbreviations

PIM: Potentially inappropriate medication
STOPP: Screening Tool for Older Persons’ Prescriptions
STOPP-J: Screening Tool for Older Persons’ Appropriate Prescriptions for Japanese
NSAID: Nonsteroidal anti-inflammatory drug
SSRI: Selective serotonin reuptake inhibitor
