# Supplementary material for: Effect of number of medications and use of potentially inappropriate medications on frailty among early-stage older outpatients
Source: J Pharm Health Care Sci. 2021 May 3;7:15. doi: 10.1186/s40780-021-00195-x (PMC8091752; doi:10.1186/s40780-021-00195-x)
Supplement: Supplementary file 1 — Additional file 1: Supplementary Table 1. Correlation Coefficients between PIM Use and Frailty Score Level. We estimated the correlation between frailty score level and PIM use for each subcategory. [file 40780_2021_195_MOESM1_ESM.docx]

**Supplementary Table 1** Correlation Coefficients between PIM Use and Frailty Score Level

| Category | Subcategory | *n* | Correlation coefficient^a^ | *p-*value |
| --- | --- | --- | --- | --- |
| Antipsychotics | Any antipsychotic drugs | 0 | NA | NA |
| Hypnotics | Benzodiazepines^b^ | 90 | 0.114 | < 0.001 |
|  | Non-benzodiazepine hypnotics | 34 | 0.037 | 0.258 |
| Antidepressants | Tricyclic antidepressants | 2 | 0.070 | 0.033 |
|  | SSRIs | 12 | 0.069 | 0.035 |
| Sulpiride | Sulpiride | 4 | 0.055 | 0.097 |
| Antiparkinson drugs | Antiparkinson drugs (only anticholinergic drugs) | 1 | 0.036 | 0.269 |
| Steroids | Corticosteroids (oral) | 30 | 0.043 | 0.195 |
| Antithrombotic drugs (antiplatelet drugs and anticoagulants) | Antiplatelet drugs including aspirin | 84 | 0.103 | 0.002 |
|  | Combined therapy with multiple antithrombotic drugs | 21 | 0.078 | 0.018 |
| Digitalis | Digoxin | 5 | -0.003 | 0.932 |
| Diuretics | Loop diuretics | 16 | 0.106 | 0.001 |
|  | Aldosterone antagonists | 11 | 0.026 | 0.425 |
| β-blockers | Non-selective β-blockers | 0 | NA | NA |
| α-blockers | α_1_-receptor blocker nonselective for receptor subtypes | 7 | 0.045 | 0.17 |
| First-generation H_1_ receptor antagonists | H_1_ receptor antagonists (first-generation) | 6 | 0.038 | 0.248 |
| H_2_ receptor antagonists | H_2_ receptor antagonists | 30 | 0.078 | 0.018 |
| Antiemetics | Antiemetic drugs | 6 | 0.023 | 0.483 |
| Laxatives | Magnesium oxide as Laxatives | 61 | -0.006 | 0.845 |
| Antidiabetic drugs | Sulfonylureas | 39 | 0.075 | 0.023 |
|  | Biguanides | 48 | 0.045 | 0.177 |
|  | Thiazolidine derivatives | 11 | 0.072 | 0.028 |
|  | α-glucosidase inhibitors | 17 | 0.004 | 0.896 |
|  | SGLT2 inhibitors | 38 | 0.003 | 0.924 |
| Insulin | Sliding-scale Insulin | 0 | NA | NA |
| Overactive bladder medications | Oxybutynin (oral) | 0 | NA | NA |
|  | Muscarinic receptor antagonists | 26 | 0.084 | 0.01 |
| NSAIDs | NSAIDs | 71 | 0.162 | < 0.001 |

NA not available; NSAIDs non-steroidal anti-inflammatory drugs; PIMs potentially inappropriate medications; SGLT2 sodium-glucose co-transporter 2; SSRIs selective serotonin reuptake inhibitors

^a^ Spearman rank correlation coefficient

^b^ Including use as antianxiety drugs
